# Treatment Outcome in Male Gambling Disorder Patients Associated with Alcohol Use

**DOI:** 10.3389/fpsyg.2016.00465

**Published:** 2016-03-31

**Authors:** Susana Jiménez-Murcia, Amparo Del Pino-Gutiérrez, Fernando Fernández-Aranda, Roser Granero, Anders Hakänsson, Salomé Tárrega, Ana Valdepérez, Neus Aymamí, Mónica Gómez-Peña, Laura Moragas, Marta Baño, Anne Sauvaget, Maria Romeu, Trevor Steward, José M. Menchón

**Affiliations:** ^1^Department of Psychiatry, Bellvitge University Hospital-IDIBELLBarcelona, Spain; ^2^Ciber Fisiopatología Obesidad y Nutrición, Instituto de Salud Carlos IIIBarcelona, Spain; ^3^Departament of Clinical Sciences, School of Medicine, University of BarcelonaBarcelona, Spain; ^4^Nursing Department of Mental Health, Public Health, Maternal and Child Health, Nursing School, University of BarcelonaBarcelona, Spain; ^5^Departament de Psicobiologia i Metodologia, Universitat Autònoma de BarcelonaBarcelona, Spain; ^6^Division of Psychiatry, Department of Clinical Sciences, Lund UniversityLund, Sweden; ^7^Department of Psychiatry, Hospital de la Santa Creu i Sant PauBarcelona, Spain; ^8^Addictology and Psychiatry Department, Nantes University HospitalNantes, France; ^9^CIBER Salud Mental, Instituto Carlos IIIBarcelona, Spain

**Keywords:** alcohol abuse, gambling disorder, personality, treatment response, at-risk drinking

## Abstract

**Aims:** The primary objective of this study was to analyze the association between alcohol consumption and short-term response to treatment (post intervention) in male patients with gambling disorder enrolled in a group cognitive behavioral therapy (CBT) program.

**Methods**: The sample consisted of 111 male individuals with a diagnosis of Gambling Disorder, with a mean age of 45 years (*SD* = 12.2). All participants were evaluated by a comprehensive assessment battery and assigned to CBT groups of 10–14 patients attending 16 weekly outpatient sessions lasting 90 min each.

**Results**: The highest mean pre- and post-therapy differences were recorded for the alcohol risk/dependence group on the obsessive/compulsive and anxiety dimensions of the SCL-90-R. As regards the presence of relapses and dropouts over the course of the CBT sessions, the results show a significant association with moderate effect size: patients with risk consumption or alcohol dependence were more likely to present poor treatment outcomes.

**Conclusions:** Alcohol abuse was frequent in GD, especially in patients with low family income and high accumulated debts. High levels of somatization and high overall psychopathology (measured by the SCL-90-R) were associated with increased risk of alcohol abuse. Alcohol abuse was also associated with poor response to treatment.

## Introduction

The general characteristics of addictive behaviors include an intense desire to satisfy a need, loss of control, and persistence in maladaptive behavior despite the negative consequences (Stinchfield et al., 2005). Given the similarities in terms of biopsychosocial vulnerability and response to treatment, gambling disorder (GD) has recently been classified as a non-substance addiction and has been included in the Diagnostic Statistical Manual (DSM-5) under the category “Substance-related and Addictive Disorders” (American Psychiatric Association, 2013).

Among the comorbidities associated with GD, studies have shown that the most frequent is substance use disorder (SUD), especially nicotine dependence (35–75%) (Dowling et al., 2015), and alcohol use disorders (AUDs) (21–65%) (el-Guebaly et al., 2006). However, few studies have systematically explored alcohol consumption in patients being treated for GD using validated measurement instruments. Data obtained from clinical interviews suggest that between 76% (Grant and Grosz, 2004) and 100% (Echeburúa et al., 2011) of individuals in treatment for GD report problems related to alcohol consumption. Studies of alcohol consumption during gambling episodes reveal riskier betting patterns and negative consequences (Ellery et al., 2005; Phillips and Ogeil, 2007; French et al., 2008).

As for psychological therapies, cognitive-behavioral therapy (CBT) has proved to be superior to no treatment and effective in the short and medium term (Rash and Petry, 2014), obtaining rates of abstinence from gambling during the first months of follow-up of around 70% (Jiménez-Murcia et al., 2007, 2015; Dowling et al., 2015). In relation to relapse, some authors define it as a violation of the goals set with respect to gambling behavior (Hodgins and El-Guebaly, 2004), while others argue that a single gambling episode should not be considered a relapse unless it is associated with a feeling of loss of control (Blaszczynski et al., 1991). Previous studies identified relapse rates of between 12% (Aragay et al., 2015) and 50% (Ledgerwood and Petry, 2006a). The rates of dropout from treatment are similar, ranging between 14 and 50% (Melville et al., 2007). Moreover, relapse and dropout have been associated with the fact that gambling is seen as a form of social interaction, or as a strategy to escape from personal problems or to resolve financial difficulties, or as a source of excitement or stimulation (Melville et al., 2007; Smith et al., 2010). Other research also describes the involvement of clinical, psychopathological and treatment characteristics such as early age of onset of GD and short duration (Petry, 2003), and motivation and adherence to the guidelines prescribed between sessions (Smith et al., 2010; Dunn et al., 2012; Gómez-Peña et al., 2012; Jimenez-Murcia et al., 2012; Jiménez-Murcia et al., 2015; Ramos-Grille et al., 2015).

Regarding predictors of poor response to treatment, some sociodemographic characteristics such as younger age, low education, being single, or ethnicity have been reported (Ingle et al., 2008; Bischof et al., 2014; Jiménez-Murcia et al., 2015). The involvement of certain clinical variables has also been described, such as problems with emotional regulation (e.g., isolation, irritability, and boredom), disruption of daily activities (Hodgins and El-Guebaly, 2010), high debts and marital problems (Poirier-Arbour et al., 2014; Jiménez-Murcia et al., 2015). Other variables associated with poor response to treatment include low motivation to change, active gambling behavior at inclusion in the therapy program, or gambling small amounts of money (Gómez-Peña et al., 2012; Aragay et al., 2015). Predictors of good response to treatment identified include family and social support (Bertrand et al., 2008; Ingle et al., 2008).

Alcohol use in patients with other psychiatric disorders is widely held to have adverse effects both on the course of the disorder and on treatment outcome (Barnes et al., 2009; Kelly et al., 2012; Colpaert et al., 2013). In GD, however, contradictory results have been obtained. Previous studies of the effect of lifetime alcohol and substance abuse on treatment response found that, while these variables indicated increased severity of the gambling problem, there was no association between consumption and treatment outcome (Stinchfield et al., 2005). In contrast, Hodgins and-Guebaly found that GD relapse was 2.68 times more likely in alcohol consumers after 6 months of abstinence (Hodgins and El-Guebaly, 2010), and emphasized the importance of treating both addictions simultaneously. Similarly conflicting results are observed in studies evaluating the treatment of GD in patients who abuse substances other than alcohol (Toneatto and Brennan, 2002; Redish et al., 2007). In this study of GD patients, we assessed alcohol consumption using AUDIT, a collaborative project developed by the World Health Organization (WHO) (Saunders et al., 1993).

The study has three aims: (1) To compare two groups of GD patients with low and high AUDIT scores at baseline and post-therapy, and to assess the pre-post changes; (2) To estimate the predictive value of baseline AUDIT scores with regard to the risk of relapse and dropout during treatment; and (3) To assess the incremental predictive ability of AUDIT scores with regard to the short-term mean changes produced in patients' clinical status between the beginning and end of treatment.

## Methods

### Participants

The sample consisted of consecutive patients who attended the Pathological Gambling Unit in the Psychiatry Department of the University Hospital of Bellvitge in Barcelona (Spain). This public hospital is certified as a tertiary center for the treatment of GD and oversees the treatment of highly complex cases. The Pathological Gambling Unit attends people with distinct behavioral addictions, such as GD, but also other behavioral addictions (the most common disorders at present are compulsive buying, technology addiction, and sex addiction). After the initial assessment process, different intervention programs are considered and applied to patients depending on their diagnosis and individual characteristics.

The sample in this study included all the consecutive male individuals with a GD diagnosis, who met inclusion criteria for the unit's group CBT treatment program, between January 2010 and June 2014. All the participants were assessed by expert clinical psychologists and psychiatrists with more than 15 years of clinical experience in the field of GD, and were diagnosed with the condition if they met the DSM-IV criteria. The patients were also evaluated face-to-face with an adapted version of the structured clinical interview for DSM-IV axis I disorders, SCID-I (First et al., 1997), which covers lifetime and current comorbid disorders. Exclusion criteria were: female sex, presence of an organic mental disorder, intellectual disability, the presence of substance use disorders at evaluation which were not being simultaneously treated as GD treatment and for which the patients were not motivated to treat, active psychotic disorder, other behavioral addictions apart from GD requiring treatment different from the aforementioned CBT group. Final sample for the analyses included *n* = 111 participants.

The present study was carried out in accordance with the latest version of the Declaration of Helsinki. The University Hospital of Bellvitge Ethics Committee of Clinical Research approved the study, and written informed consent was obtained from all participants.

### Instruments

A comprehensive assessment battery was administered to measure GD. Alcohol consumption was assessed by the AUDIT method described below. Additional demographic, clinical, and social/family variables related to gambling were measured using a semi-structured, face-to-face clinical interview described elsewhere (Jiménez-Murcia et al., 2007).

*South Oaks Gambling Screen* (SOGS) (Lesieur and Blume, 1987; Spanish validation by Echeburúa et al., 1994). This is a 20-item diagnostic questionnaire that discriminates between probable pathological gamblers, problematic gamblers and non-problematic gamblers. The Spanish validation of this questionnaire shows high reliability and validity. Test-retest reliability is 0.98 (*p* < 0.001) and internal consistency 0.94 (Cronbach's alpha). The convergent validity with respect to DSM-III-R criteria for pathological gambling (American Association Psychiatric, 1987) was estimated to be 0.92. In this study the total score was used.

*Diagnostic questionnaire for Pathological Gambling according to DSM-IV criteria* (Stinchfield, 2003), Spanish adaptation by Jimenez-Murcia et al. (2009). This 19-item questionnaire assesses the DSM-IV diagnostic criteria for PG. Internal consistency ranged between 0.81 for the general population and 0.77 for gambling treatment samples. Convergent validity with the SOGS scores was very good: *r* = 0.77 for the general population and *r* = 0.75 for gambling treatment groups (Stinchfield, 2003).

*Alcohol Use Disorders Identification Test* (AUDIT) (Saunders et al., 1993). This test was developed as a simple screening method for excessive alcohol consumption. The AUDIT consists of 10 questions about the level of consumption, symptoms of dependence, and alcohol-related consequences. Internal consistency has been found to be high, and rest-retest data have suggested high reliability (0.86) and sensitivity of approximately 0.90. Specificity in different settings and for different criteria averages 0.80 or more (Martínez, 1996). Here, cut-off points of 8 and 20 were used to identify individuals with alcohol abuse and alcohol dependence respectively (Reinert, 2002).

*Symptom Check List-90 items-Revised* (SCL-90-R; Derogatis, 1994; Spanish validation by González de Rivera, 2001). The SCL-90-R was administered to evaluate a broad range of psychological problems and symptoms of psychopathology. This test measures nine primary symptom dimensions: somatization, obsession-compulsion, interpersonal sensitivity, depression, anxiety, hostility, phobic anxiety, paranoid ideation, and psychoticism. It also includes three global indices: a global severity index (GSI), which measures overall psychological distress; a positive symptom distress index (PSDI) to measure the intensity of symptoms; and a positive symptom total (PST), which reports the total self-reported symptoms. The GSI can be used as a summary of the test. This scale has been validated in a Spanish population, obtaining an internal consistency (alpha coefficient) for the items ranging between 0.81 and 0.90.

*Other socio-demographic and clinical variables*. Additional demographic, clinical, and social/family variables related to gambling were measured using a semi-structured face-to-face clinical interview described elsewhere (Jiménez-Murcia et al., 2006). Among the gambling behavior variables covered were the number of previous treatment attempts, the type of problem gambling, whether there was more than one preferred form of gambling, the age of onset of gambling and of gambling-related problems, the average and the maximum amounts bet in a single episode, and the total accumulated debts. In addition, the interview explored certain maintaining factors such as gambling to chase losses or to avoid negative emotional states, magical thinking and the illusion of control, ritualistic behavior, characteristics of the last gambling episode prior to the visit to the unit, and the family situation generated by the gambling problems.

### Procedure

All participants were interviewed and evaluated by clinical psychologists and physicians with more than 15 years experience in the diagnosis and treatment of these disorders. Throughout the treatment period, attendance, the control of spending and gambling behavior, compliance with treatment (subjectively rated by the therapist as good, fair or poor) and occurrence of relapses were recorded on an observation sheet. Patients were also instructed to perform tasks in preparation for the following session. The observation sheet was completed during the treatment session by the therapist and by a clinically-trained co-therapist. Moreover, at the end of the last session of treatment (in which patients finalized the CBT program), we conducted a post-treatment assessment session, which consisted of the administration of the SOGS and the SCL-90-R (on both self-report tests, patients had to answer items considering their current state). The time for completion of the two questionnaires was not more than 30 min. For work reasons, most patients reported preferring to complete this post-treatment evaluation during the same visit as opposed to having to return to the unit after a few days.

### Treatment

Patients were assigned to outpatient CBT groups comprising 10–14 patients each. The CBT group therapy consisted of 16 weekly outpatient sessions lasting 90 min each and a follow-up period lasting up to 2 years, though the current study assesses data from the first 3 months of the follow-up only. A family member (usually the spouse or partner) was involved in the treatment. Family members attended seven of the 16 weekly treatment sessions and the entire follow-up period. Their functions were to help and support the patient, to acquire a better understanding of the disorder, to manage situations of risk, and to help patients regain their confidence. Family members who attended group therapy also collaborated in some of the treatment techniques such as stimulus control (initial control of money) and in helping patients to find alternative activities to gambling such as hobbies and healthy distractions.

Each group was led by an experienced clinical psychologist with the aid of a clinically trained co-therapist. The goal of the treatment was to train patients to implement CBT strategies in order to achieve full recovery, defined as full abstinence from all types of gambling behavior. The general topics addressed in the therapy sessions included: psychoeducation regarding the disorder (its course, vulnerability factors, diagnostic criteria, biopsychosocial models of GD, phases, etc.), stimulus control (money management, avoidance of risk situations, self-exclusion program, changing risky routes, etc.), response prevention (alternative and compensatory behaviors), cognitive restructuring focused on the illusions of control over gambling and magical thinking, reinforcement and self-reinforcement, skills training, and relapse prevention techniques. This treatment program has already been described elsewhere (Jiménez-Murcia et al., 2006) and its short- and medium-term effectiveness has also been reported (Jiménez-Murcia et al., 2007, 2015; Jimenez-Murcia et al., 2012).

Moreover, this treatment protocol contemplates that those patients who have alcohol use disorder or another substance use disorder (or even if they do not meet all the criteria for diagnosis, but have a pattern of hazardous drinking behavior) should also specifically undergo alcohol and drug treatment at their corresponding primary care center within the public health network. If patients do not present a sufficient level of motivation, the treatment applied for them is individual outpatient therapy. That is to say, individual CBT has similar characteristics to group CBT but, in addition to therapy for their problem gambling, includes a module of motivational sessions, based on motivational interviewing Miller and Rollnick (2013), to facilitate the recognition of their alcohol problem and to draw them to use the above-mentioned resources.

To ensure treatment fidelity, therapists adhered closely to the treatment manual. The two therapists in charge of the treatment groups (M.N.A. and M.G.-P.) have more than 15 years of experience of running group and individual CBT therapy for individuals with GD. Weekly case discussions were held between the therapist, the clinically trained co-therapist, and the rest of the team at the unit.

### Statistical methods

Analyses were carried out with SPSS20 for Windows. Since other psychiatric conditions are usually considered in both a dimensional and a categorical approach in clinical and research areas, alcohol measures were handled likewise. The AUDIT measure was analyzed on a dimensional scale (AUDIT-raw-total score) and a binary categorization null/low risk for individuals with an AUDIT score between 0 and 7 and risk/dependence for an AUDIT score of 8 or higher). Results obtained for the dimensional AUDIT-raw-total score will be interpreted as the measure of the *individuals' alcohol use*, while the AUDIT-binary-group is a categorization of an *individuals' risk leve*l.

Firstly, the comparison of the clinical measures between the two AUDIT-risk-groups was carried out with χ^2^ tests for categorical variables and with *t*-test procedures for quantitative data. Simultaneously Pearson's correlation estimated the association between the alcohol level in a dimensional scale (AUDIT raw total) and the psychometrical measures (|*r*| > 0.30 was considered good effect size).

Secondly, binary logistic regressions measured the contribution of the AUDIT measures (considering both perspectives, the binary categorization AUDIT-risk-group and the dimensional AUDIT-raw-total) on the primary treatment responses (dropout, relapse and compliance). Goodness-of-fit was measured through the Hosmer-Lemeshow test and the global predictive-discriminative capacity with Nagelkerke's pseudo-R^2^.

Thirdly, Kaplan-Meier functions estimated the cumulative survival function by measuring the time to the presence of relapse and dropout during the development of the therapy. The Kaplan-Meier estimator is a procedure which is included in survival analysis techniques to estimate the survival function from lifetime data, that is, the proportion of subjects remaining without the presence of a specific outcome during the follow-up. In this study, this function estimated the length of time patients remained without relapse (“survive”) during the treatment.

The Simes' correction method was used to control Type-I error due to multiple statistical comparisons (Simes, 1986). This procedure was included into the Familywise error rate stepwise procedures, and offers a more powerful test than the classical Bonferroni correction. In addition, since effect sizes are the relevant objective of the analyses (*p*-values are strongly dependent to sample sizes), all effect sizes for the relationships analyzed in this study have been estimated: Cohen's-d coefficient for mean comparisons (|d| < 0.5 was considered poor, |d| > 0.5 was considered moderate and |d| > 0.8 was considered large) and 95%CI for the parameter estimators in the linear and logistic regressions.

## Results

### Sample description

Table 1 includes descriptions of the total sample and is stratified according to alcohol level group. Around half the patients had finished elementary (58.7%) or high school (35.6%) education, most were married (53.3%) and half were employed (50.9%). Mean age was 45 years (*SD* = 12.2). The mean duration of the disorder was 5.38 years (*SD* = 5.0). Slot-machines were the main gambling problem for most participants (82.9%). Eighty-nine patients (80.2%) were classified in the null/low alcohol group and 22 (19.8%) in the risk/dependence group. Table 1 displays the descriptions for the total sample of the study (*n* = 111), and the comparison of the socio-demographical and clinical variables between the two alcohol level groups. No statistical differences were found between the two groups with regard to many of the variables presented in Table 1, except for the prevalence of patients who reported bingo gambling (with a higher proportion for the risk/dependence group; χ^2^ = 11.9, *df* = 1, *p* = 0.008) and the mean total accumulated debts (with higher means also reported for the risk/dependence group; *t* = 2.47, *df* = 109, *p* = 0.045).

**Table 1:** **Sample description**.

		**Total sample (*n =* 111)**	**Null/low (*n* = 89)**	**Risk/dependence (*n* = 22)**	*p
Education; %	Primary	58.7%	54.2%	76.2%	0.353
	Secondary	35.6%	38.6%	23.8%	
	University	5.8%	7.2%	0%	
Employment status; %	Employed	50.9%	50.0%	54.5%	0.758
Civil status; %	Single	32.7%	31.8%	36.4%	0.703
	Married	53.3%	55.3%	45.5%	
	Divorced	14.0%	12.9%	18.2%	
Own income (euros)*;* mean–SD		1297.1–790.1	1321.9–814.6	1191.9–685.0	0.696
Family income (euros)*;* mean–SD		2204.3–1317.2	2328.5–1397.1	1637.5–627.0	0.285
Gambling preferences; %	Slot machines	82.9%	84.3%	77.3%	0.823
	Bingo	4.5%	1.1%	18.2%	**0.008**
	Lotteries	5.4%	5.6%	4.5%	0.962
	Casinos	1.8%	2.2%	0%	0.782
	Cards	2.7%	2.2%	4.5%	0.823
	Sport bets	1.8%	1.1%	4.5%	0.731
	Online gambling	5.4%	5.6%	4.5%	0.879
	Other	0.9%	1.1%	0%	0.782
Comorbid mental disorder (present)		21.6%	20.2%	27.3%	0.734
Comorbid mental disorder (past)		34.2%	32.6%	40.9%	0.957
Tobacco use		58.6%	60.7%	50.0%	0.734
Substances abuse		9.1%	8.0%	13.6%	0.957
Age (years–old)*;* mean–SD		45.0–12.2	44.6–12.4	46.4–11.8	0.869
Evolution of gambling (years)*;* mean–SD		5.38–5.0	5.34–5.0	5.57–5.0	0.957
Age of onset (years-old)*;* mean–SD		39.09–12.7	39.24–12.6	38.29–13.7	0.869
Maximum money–per episode*;* mean–SD		609.8–1128.8	624.1–1210.9	551.8–724.9	0.903
Mean money spent–per episode*;* mean–SD		109.4–229.7	110.2–246.3	106.4–149.1	0.945
Total accumulated debts*;* mean–SD		12000.8–41591	7270.7–16074	31136.4–86602	**0.045**

*p (Bold, significant comparison).

### Comparison of gambling severity and general psychopathology

The first part of Table 2 displays the comparison between patients in the two alcohol level groups (null/low vs. risk/dependence) for the pre-therapy SOGS-total and SCL-90 scores, the post-therapy values and the pre-post changes. At the beginning of the study (baseline state), no statistical differences emerged comparing these clinical measures, although moderate effect size differences were obtained for the SCL-90-R somatization and the PST scales.

**Table 2:** **Comparison at baseline, post-therapy, and pre-post changes**.

	**AUDIT-group**							**AUDIT**	
	**Null/low; (*****n*** = **89)**		**Risk/dep.; (*****n*** = **22)**		**Mean comparison**			**raw-total**	
	**Mean**	**SD**	**Mean**	**SD**	**T-stat**	***p***	**|*d*|**	***r***	***p***
**BASELINE VALUES**									
SOGS-total score	10.03	3.00	11.30	3.18	1.678	0.171	0.41	0.145	0.280
SCL-90: Somatization	0.88	0.68	1.19	0.59	1.936	0.134	**0.50**^**†**^	0.143	0.280
SCL-90: Obses./comp.	1.06	0.66	1.37	0.69	1.885	0.264	0.45	0.198	0.174
SCL-90: Interpersonal	0.95	0.76	1.25	0.68	1.638	0.171	0.41	0.137	0.299
SCL-90: Depressive	1.49	0.81	1.70	0.84	1.062	0.364	0.26	0.102	0.418
SCL-90: Anxiety	0.91	0.63	1.21	0.67	1.956	0.134	0.47	0.192	0.174
SCL-90: Hostility	0.85	0.75	1.10	0.87	1.363	0.254	0.32	0.143	0.280
SCL-90: Phobic anxiety	0.40	0.46	0.52	0.48	1.025	0.364	0.25	0.127	0.318
SCL-90: Paranoia	0.86	0.73	0.96	0.71	0.554	0.581	0.14	0.055	0.662
SCL-90: Psychotic	0.87	0.66	1.20	0.80	2.017	0.134	0.46	0.169	0.205
SCL-90: GSI score	1.01	0.58	1.27	0.54	1.897	0.264	0.47	0.172	0.205
SCL-90: PST score	46.66	19.66	58.67	16.08	2.590	0.143	**0.67**^**†**^	0.259	0.071
SCL-90: PSDI score	1.85	0.52	1.93	0.58	0.655	0.557	0.15	0.041	0.754
**POST-THERAPY VALUES**									
SOGS-total score	1.89	2.32	3.20	4.72	1.558	0.377	0.35	**0.346**^**†**^	0.043*
SCL-90: Somatization	0.43	0.53	0.55	0.49	0.972	0.643	0.24	0.190	0.174
SCL-90: Obses./comp.	0.57	0.61	0.63	0.67	0.442	0.779	0.10	0.217	0.145
SCL-90: Interpersonal	0.43	0.50	0.54	0.70	0.793	0.658	0.17	0.179	0.182
SCL-90: Depressive	0.59	0.58	0.70	0.64	0.806	0.590	0.19	0.202	0.174
SCL-90: Anxiety	0.38	0.46	0.40	0.46	0.224	0.865	0.05	0.147	0.280
SCL-90: Hostility	0.36	0.50	0.42	0.62	0.533	0.764	0.12	0.254	0.071
SCL-90: Phobic anxiety	0.18	0.38	0.20	0.25	0.249	0.842	0.07	0.032	0.782
SCL-90: Paranoia	0.46	0.61	0.56	0.54	0.703	0.662	0.17	0.181	0.182
SCL-90: Psychotic	0.34	0.46	0.40	0.56	0.501	0.764	0.11	0.191	0.174
SCL-90: GSI score	0.45	0.44	0.53	0.51	0.739	0.592	0.17	0.225	0.136
SCL-90: PST score	25.07	18.62	31.68	19.09	1.485	0.377	0.35	**0.300**^**†**^	0.043*
SCL-90: PSDI score	1.45	0.49	1.35	0.38	0.922	0.643	0.24	0.004	0.966
**PRE-POST CHANGES**									
SOGS-total score	8.84	2.83	8.95	2.70	0.170	0.865	0.04	0.006	0.966
SCL-90: Somatization	0.48	0.64	0.71	0.62	1.522	0.377	0.37	0.064	0.638
SCL-90: Obses./comp.	0.52	0.71	0.86	0.64	2.045	0.332	**0.50**^**†**^	0.128	0.318
SCL-90: Interpersonal	0.54	0.71	0.80	0.85	1.467	0.377	0.33	0.095	0.432
SCL-90: Depressive	0.94	0.78	1.11	0.72	0.970	0.643	0.24	0.059	0.659
SCL-90: Anxiety	0.57	0.62	0.89	0.66	2.171	0.332	**0.51**^**†**^	0.165	0.205
SCL-90: Hostility	0.50	0.68	0.79	0.73	1.715	0.377	0.40	0.090	0.453
SCL-90: Phobic anxiety	0.24	0.42	0.33	0.40	0.897	0.643	0.22	0.102	0.415
SCL-90: Paranoia	0.43	0.70	0.46	0.81	0.171	0.865	0.04	−0.032	0.782
SCL-90: Psychotic	0.55	0.63	0.89	0.80	2.128	0.332	0.47	0.122	0.318
SCL-90: GSI score	0.59	0.56	0.83	0.56	1.835	0.359	0.44	0.104	0.415
SCL-90: PST score	22.36	21.38	29.29	18.79	1.391	0.395	0.34	0.056	0.662
SCL-90: PSDI score	0.41	0.48	0.65	0.56	1.978	0.332	0.45	0.125	0.318

SD, standard deviation. ^*^Bold, significant parameter (0.05 level).

*^†^Bold, effect size in the moderate to high range (|*d*| > 0.50 or |r| > 0.30)*.

No statistical differences were recorded for the SOGS-total and the SCL-90 mean scores at the end of the treatment (post-therapy values), and effect sizes of the mean differences were in the low range (*d*-coefficients clearly lower than 0.50).

Considering pre-post changes, statistically no differences emerged. However, from a practical-clinical perspective, higher mean differences were recorded for the alcohol risk/dependence group on the SCL-90 obsessive/compulsive and anxiety scales (effect size in the moderate range, *d* around 0.50).

The second part of Table 2 contains the Pearson's correlation estimating the association between the dimensional AUDIT-raw-total score and the psychometrical measures considered in this study. No significant or relevant correlations emerged for the SOGS and the SCL-90R baseline measures, nor for the pre-post differences. The only significant associations were obtained for the post-therapy SOGS and SCL-90R PST scales: the higher the AUDIT-raw-total score, the higher the dysfunction registered in both these scales.

### Comparison of treatment outcome: Attendance, compliance, relapses, and dropout

Table 3 displays the comparison of the treatment outcomes between patients in the two alcohol level groups: poor attendance (missing at least three sessions), poor compliance with therapy guidelines, the presence of relapses and dropout. The results show a significant association with moderate effect size: patients with alcohol risk/dependence had a higher risk of poor treatment outcomes.

**Table 3:** **Comparison of the therapy outcomes for the AUDIT-binary-group**.

	**Null/low**	**Risk/dep**.	**Cohen's**	**Logistic regression**							
	**(*n* = 89)**	**(*n* = 22)**	***|d|***	**B**	**SE**	**$\chi_{\left(df=1\right)}^{2}$**	***p***	**OR**	**95%CI (OR)**		**N-R^2^**
Poor attendance	20.2%	54.6%	0.759*	1.555	0.503	9.554	**0.006**	4.73	1.77	12.69	0.120
Poor compliance	32.6%	59.1%	0.552*	1.095	0.489	5.011	**0.025**	2.99	1.15	7.79	0.061
Relapses	18.0%	50.0%	0.718*	1.518	0.508	8.929	**0.006**	4.56	1.69	12.35	0.114
Dropout	1.1%	18.2%	0.605*	2.973	1.148	6.713	**0.013**	19.6	2.06	185.4	0.252

^*^Bold, effect size in the moderate (|*d*| > 0.50) to high range (|d| > 0.80).

Table 4 includes the multiple logistic regressions exploring the discriminative capacity of the main sociodemographic variables of the sample (age, education, civil status, employment status) and the alcohol group (risk/dependence vs. null/low) on the four treatment outcomes considered in the study. Even after including the patients' age, education and marital and employment status, the AUDIT-group obtained significant discriminative capacity for the outcomes. Specifically: (a) patients in employment with AUDIT-risk scores were more likely to attend fewer CBT sessions; and (b) patients with AUDIT-risk scores were more likely to present poor compliance, relapse and dropout.

**Table 4:** **Logistic models measuring the contribution of the AUDIT-binary-group on the therapy primary outcomes**.

	**B**	**SE**	**$\chi_{\left(df=1\right)}^{2}$**	***p***	**OR**	**95%CI(OR)**		**N-R^2^**
**CRITERION: POOR ATTENDANCE**								
Age (years-old)	−0.007	0.022	0.102	0.749	0.99	0.95	1.04	0.250
Education (1 = primary or less vs. 0 = other)	0.236	0.526	0.202	0.653	1.27	0.45	3.55	
Marital status (1 = married vs. 0 = other)	−0.382	0.503	0.574	0.449	0.68	0.25	1.83	
Employment (1 = Employed vs. 0 = other)	1.377	0.539	6.533	**0.011**	3.96	1.38	11.40	
Audit-binary-group (1 = risk/dep. Vs. 0 = null/low)	1.727	0.578	8.913	**0.003**	5.62	1.81	17.47	
**CRITERION: POOR COMPLIANCE**								
Age (years–old)	−0.024	0.020	1.489	0.222	0.98	0.94	1.01	0.181
Education (1 = primary or less vs. 0 = other)	0.481	0.477	1.019	0.313	1.62	0.64	4.12	
Marital status (1 = married vs. 0 = other)	−0.498	0.458	1.182	0.277	0.61	0.25	1.49	
Employment (1 = Employed vs. 0 = other)	0.884	0.466	3.598	0.058	2.42	0.97	6.03	
Audit-binary-group (1 = risk/dep. Vs. 0 = null/low)	1.077	0.543	3.944	**0.047**	2.94	1.01	8.51	
**CRITERION: RELAPSES**								
Age (years–old)	−0.006	0.021	0.071	0.790	0.99	0.95	1.04	0.179
Education (1 = primary or less vs. 0 = other)	0.757	0.539	1.972	0.160	2.13	0.74	6.13	
Marital status (1 = married vs. 0 = other)	−0.181	0.502	0.130	0.719	0.83	0.31	2.23	
Employment (1 = Employed vs. 0 = other)	0.740	0.514	2.078	0.149	2.10	0.77	5.74	
Audit-binary-group (1 = risk/dep. vs. 0 = null/low)	1.429	0.546	6.859	**0.009**	4.17	1.43	12.16	
**CRITERION: DROPOUT**								
Age (years–old)	0.041	0.044	0.873	0.350	1.04	0.96	1.14	0.366
Education (1 = primary or less vs. 0 = other)	−0.524	1.207	0.188	0.664	0.59	0.06	6.31	
Marital status (1 = married vs. 0 = other)	−0.351	1.101	0.102	0.749	0.70	0.08	6.08	
Employment (1 = Employed vs. 0 = other)	−1.594	1.258	1.606	0.205	0.20	0.02	2.39	
Audit-binary-group (1 = risk/dep. vs. 0 = null/low)	3.132	1.259	6.194	**0.013**	22.93	1.95	270.2	

*Bold, significant parameter in the logistic regression. N-R^2^, Nagelkerke's pseudo R^2^*.

Table 5 contains again the logistic regressions considering the dimensional AUDIT-raw-total score as a predictor of the primary CBT outcomes. Results were similar to those obtained for the binary AUDIT-risk-group for the criteria poor attendance and dropout. However, no significant contribution of the dimensional AUDIT-raw-total was obtained on the criteria poor compliance and relapses.

**Table 5:** **Logistic models measuring the contribution of the AUDIT-raw-total score on primary therapy outcomes**.

	**B**	**SE**	**$\chi_{\left(df=1\right)}^{2}$**	***p***	**OR**	**95%CI(OR)**		**A-R^2^**
**CRITERION: POOR ATTENDANCE**								
Age (years–old)	0.001	0.021	0.002	0.968	1.00	0.96	1.04	0.213
Education (1 = primary or less vs. 0 = other)	0.217	0.519	0.175	0.676	1.24	0.45	3.43	
Marital status (1 = married vs. 0 = other)	−0.522	0.494	1.116	0.291	0.59	0.23	1.56	
Employment (1 = Employed vs. 0 = other)	1.407	0.532	6.998	**0.008**	4.08	1.44	11.58	
Audit-binary-group (1 = risk/dep. vs. 0 = null/low)	0.107	0.044	6.028	**0.014**	1.11	1.02	1.21	
**CRITERION: POOR COMPLIANCE**								
Age (years–old)	−0.020	0.019	1.029	0.310	0.98	0.94	1.02	0.154
Education (1 = primary or less vs. 0 = other)	0.499	0.477	1.096	0.295	1.65	0.65	4.20	
Marital status (1 = married vs. 0 = other)	−0.573	0.453	1.598	.206	0.56	0.23	1.37	
Employment (1 = Employed vs. 0 = other)	0.905	0.460	3.865	**0.049**	2.47	1.00	6.09	
Audit-binary-group (1 = risk/dep. vs. 0 = null/low)	0.054	0.040	1.790	0.181	1.06	0.98	1.14	
**CRITERION: RELAPSES**								
Age (years–old)	0.000	0.021	0.000	0.989	1.00	0.96	1.04	0.118
Education (1 = primary or less vs. 0 = other)	0.796	0.531	2.244	0.134	2.22	0.78	6.28	
Marital status (1 = married vs. 0 = other)	−0.301	0.488	0.381	0.537	0.74	0.28	1.93	
Employment (1 = Employed vs. 0 = other)	0.775	0.500	2.404	0.121	2.17	0.81	5.79	
Audit-binary-group (1 = risk/dep. vs. 0 = null/low)	0.062	0.041	2.281	0.131	1.06	0.98	1.15	
**CRITERION: DROPOUT**								
Age (years–old)	0.068	0.048	1.989	0.158	1.07	0.97	1.18	0.269
Education (1 = primary or less vs. 0 = other)	−0.375	1.112	0.114	0.736	0.69	0.08	6.08	
Marital status (1 = married vs. 0 = other)	−0.930	1.023	0.825	0.364	0.39	0.05	2.93	
Employment (1 = Employed vs. 0 = other)	−1.066	1.231	0.750	0.387	0.34	0.03	3.84	
Audit-binary-group (1 = risk/dep. vs. 0 = null/low)	0.150	0.068	4.842	**0.028**	1.16	1.02	1.33	

*Bold, significant parameter in the logistic regression. N-R^2^, Nagelkerke's pseudo R^2^*.

**Figure 1:**
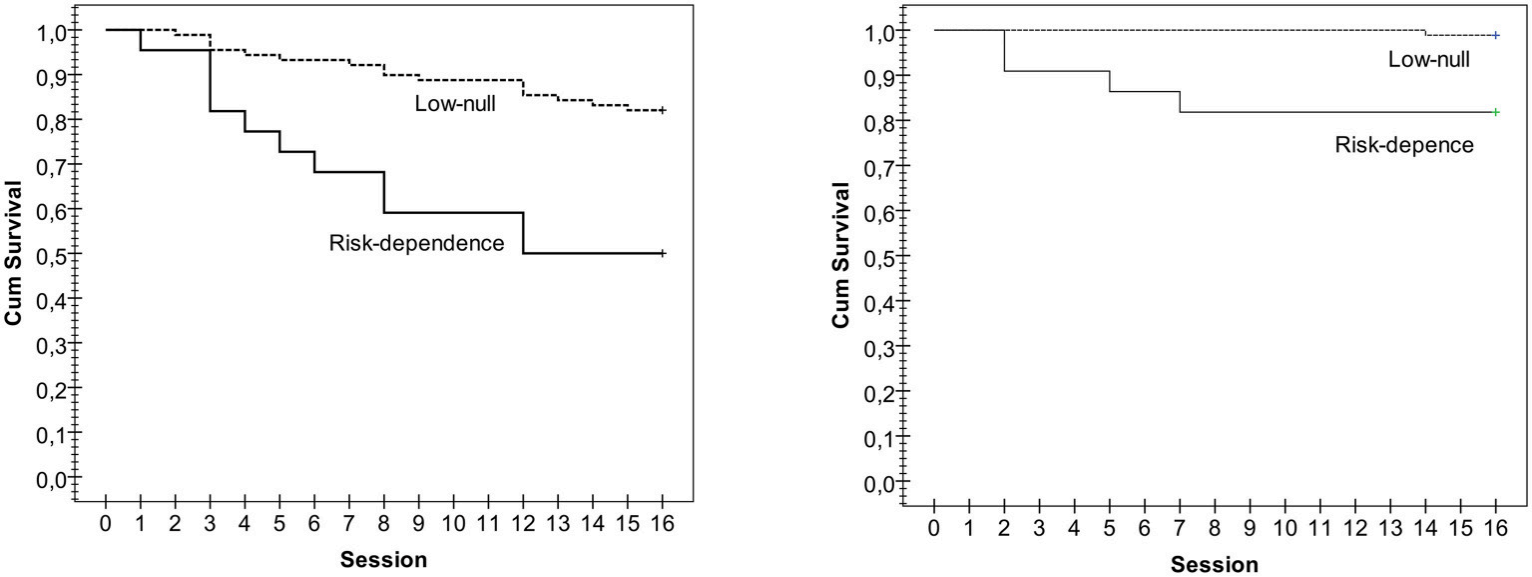
**Kaplan-Meier functions for the survival time to the first relapse and dropout during therapy stratified by AUDIT-group**.

The first section of Figure 1 includes the cumulative-survival functions (Kaplan-Meier estimation) for time to first relapse during therapy. Overall comparison for the AUDIT-binary classification (low/null vs. risk/dependence) achieved significant results for the three chi-squared tests: Log-Rank (Mantel-Cox χ^2^ = 12.21, *p* = 0.001), Breslow (Generalized Wilcoxon χ^2^ = 12.76, *p* < 0.001) and Tarone-Ware (χ^2^ = 12.51, *p* = 0.001): relapses occurred rapidly in patients in the risk/dependence group. After the fourth therapy session, 23% of the patients who recorded risk/dependence scores in the AUDIT at the baseline had relapsed; after eight sessions 40% reported relapses and at the end of the twelfth session all the relapses had been recorded (the total relapse rate was 50%). In the null/low group at baseline, 5.4% of patients had relapsed during the first four sessions, 10% between sessions 1 and 8 and 15% between sessions 1 and 12 (the other 3% of relapses were recorded between sessions 12 and 15).

The second section in Figure 1 displays the Kaplan-Meier functions for the time to dropout during therapy. Chi-squared tests showed significant comparisons for the survival time to dropout between patients with null/low and risk alcohol levels: Log-Rank (Mantel-Cox χ^2^ = 12.62, *p* = 0.001), Breslow (Generalized Wilcoxon χ^2^ = 12.86, *p* < 0.001), and Tarone-Ware (χ^2^ = 12.69, *p* = 0.001): dropouts occurred rapidly in patients in the risk/dependence level group. The only dropout registered in the null/low level occurred at the end of therapy (at session 14). Dropouts in alcohol risk/dependence patients occurred after sessions 2 (cumulative survival equal to 90.9%), 5 (cumulative survival 86.4%), and 7 (cumulative survival 81.8%).

## Discussion

The main objective of this study was to analyze the association between alcohol use and the short-term response (changes between the beginning and the end of the therapy) to a CBT group program in individuals diagnosed with GD who consecutively attended a specialist GD unit. It also aimed to compare the demographic and clinical characteristics between patients who reported zero or low alcohol consumption and those with risk consumption or dependence, as well as the relationship between alcohol consumption and psychopathology.

Consistent with previous reports, the majority of subjects who sought treatment for GD were male slot-machine players (Jiménez-Murcia et al., 2015; Kessler et al., 2008; Penelo et al., 2012; Granero et al., 2015). However, patients whose main gambling problem was bingo presented higher rates of risk consumption or alcohol dependence, and higher accumulated debts. These results coincide with other studies which have suggested that players seeking to avoid negative emotions usually choose games of chance or non-strategy games such as bingo (Potenza et al., 2001; Ledgerwood and Petry, 2006a; Moragas et al., 2015). Similarly, studies on risk alcohol consumption have reported that the motivation to regulate negative emotional states may be a source of vulnerability and may cause the problem to persist (Yip and Potenza, 2014; Naqvi et al., 2015).

With regard to the levels of psychopathology and emotional distress, and once again coinciding with the literature, greater alterations were observed in patients with GD and risk alcohol consumption or dependence (Baldo et al., 2006; Yau and Potenza, 2015). These patients presented higher scores on all subscales of the SCL-90-R than GD patients with zero or low alcohol consumption; the most significant differences were found in the somatization dimension, and these patients also presented more severe general psychopathology. These dimensions reflect the presence of distress associated with physical dysfunctions and a greater intensity in the perception of health problems. Other studies have also reported higher levels of alteration and impairment in patients with GD who present comorbidity with other disorders (Poirier-Arbour et al., 2014; Dowling et al., 2015; Jiménez-Murcia et al., 2015).

As for the improvements in the clinical status of patients over the course of treatment, in agreement with previous studies we found CBT to be an effective therapy for GD, both for restoring self-control over gambling behavior (measured through the DSM-IV and SOGS) and for reducing the levels of emotional distress associated with the addiction (measured by the SCL-90-R; Jiménez-Murcia et al., 2007; Parhami et al., 2014). Among other interesting findings were the significant changes in patients with risk alcohol consumption or alcohol dependence on the dimensions of obsession/compulsion and anxiety following treatment; that is, these patients presented more changes in characteristics such as worry and rumination over problems, anxiety, stress, and fear.

In view of the high prevalence of alcohol problems in patients with GD and the limitations of the response to CBT, it seems especially important to explore the comorbidity between these two disorders in order to design and implement treatments that address both addictions (Toneatto and Brennan, 2002; Barnes et al., 2015). Some authors contend that specific treatment applied to one of the disorders may also be beneficial for the other (Yau and Potenza, 2015); indeed, it is possible that the decision to seek treatment to solve a specific problem can encourage the person to make changes in other behaviors in order to improve their health (Hall and Rossi, 2008; Noar et al., 2008). However, in some cases one addiction may be substituted for another; that is, when control over a particular addictive behavior is achieved, the frequency of the other one, which until then had not been problematic, may increase (Toneatto and Brennan, 2002).

Moreover, as regards the risk of relapse and dropout, studies of patients with other psychiatric disorders have shown that alcohol consumption has adverse effects on therapy (Barnes et al., 2009; Kelly et al., 2012; Colpaert et al., 2013). Our results also show that problem use of alcohol in patients diagnosed with GD increases the likelihood of poor response to CBT, thus corroborating previous reports (Staiger et al., 2013). Although, it was not possible to obtain information as to why patients dropped out of the treatment program in this present study, other recent studies conducted with patients from the same geographical area, and mostly with the same type of gambling problem (slot machines), have found that socio-demographic variables such as young age, lower education level and civil status (Aragay et al., 2015; Jiménez-Murcia et al., 2015), as well as high levels of neuroticism, impulsivity and novelty seeking (Aragay et al., 2015; Ramos-Grille et al., 2015) are associated with high dropout risk. Other international studies emphasize that the patient's motivation level, the number of relapses, medical or family issues, and even aspects related to treatment or the patient's therapist could be associated to treatment dropout (Melville et al., 2007). With respect to variables that are linked to relapses, optimism about one's chances of winning, difficulty tolerating boredom, lack of structure during one's leisure time and, gambling-related urges and cognitions (Ledgerwood and Petry, 2006b) or associated psychopathology severity levels (Jiménez-Murcia et al., 2015), could all be risk factors for relapse.

In conclusion, our findings show that alcohol abuse is a common condition in GD and is associated with low household income and high debts. From a psychopathological point of view, the symptomatological dimensions of somatization and high levels of overall emotional distress were associated with increased risk of alcohol abuse. An association was also identified between alcohol abuse and poor response to treatment.

Alcohol consumption in GD is a predictor of treatment failure and should be carefully assessed. Longitudinal studies are clinically important, since they allow the design of treatment programs that address the specific needs of each patient, improve adherence and response to therapy, and minimize the risk of relapse and dropout. In this sense, future research is required to estimate the long-term results of intervention programs, and should be focused on the evolution of therapy outcomes and the potential predictors of the outcomes/changes during long follow-up periods.

### Limitations

The results presented here should be considered in the light of several limitations. First, the sample used comprised consecutive patients seeking treatment at our unit; therefore, the results may not be generalizable to patients who do not seek treatment. Second, specific assessment measures for a clinical exploration, such as the structured interview for Axis II comorbid disorders, were not used. Although, an interview adapted from the SCID-I (First et al., 1997) was used to explore comorbidities, the information was gathered from the viewpoint of the clinician and was not included in the statistical analysis. Therefore, only scores on the SCL-90-R scale were considered when referring to the term “psychopathology.”

## Author contributions

All authors listed, have made substantial, direct and intellectual contribution to the work, and approved it for publication.

### Conflict of interest statement

The authors declare that the research was conducted in the absence of any commercial or financial relationships that could be construed as a potential conflict of interest.
